# Circulating MicroRNAs From Plasma Small Extracellular Vesicles as Potential Diagnostic Biomarkers in Pediatric Epilepsy and Drug-Resistant Epilepsy

**DOI:** 10.3389/fnmol.2022.823802

**Published:** 2022-02-10

**Authors:** Yilong Wang, Yeping Wang, Yi Chen, Yi Hua, Lu Xu, Mengying Zhu, Congying Zhao, Weiran Zhang, Guoxia Sheng, Liu Liu, Peifang Jiang, Zhefeng Yuan, Zhengyan Zhao, Feng Gao

**Affiliations:** ^1^Department of Neurology, Children’s Hospital, Zhejiang University School of Medicine, Hangzhou, China; ^2^Children’s Hospital, Zhejiang University School of Medicine, Hangzhou, China; ^3^National Clinical Research Center for Child Health, Children’s Hospital, Zhejiang University School of Medicine, Hangzhou, China

**Keywords:** epilepsy, microRNAs, small extracellular vesicle, anti-epileptic drug resistance, anti-epileptic drug response

## Abstract

Pediatric epilepsy is a neurological condition that causes repeated and unprovoked seizures and is more common in 1–5-year-old children. Drug resistance has been indicated as a key challenge in improving the clinical outcomes of patients with pediatric epilepsy. In the present study, we aimed to identify plasma small extracellular vesicles (sEVs) derived microRNAs (miRNAs) from the plasma samples of children for predicting the prognosis in patients with epilepsy and drug-resistant epilepsy. A total of 90 children clinically diagnosed with epilepsy [46 antiepileptic drug (AED)-responsive epilepsy and 44 drug-resistant epilepsy] and 37 healthy controls (HCs) were enrolled in this study. RNA sequencing was performed to identify plasma sEVs derived miRNAs isolated from the children’s plasma samples. Differentially expressed plasma sEVs derived miRNAs were identified using bioinformatics tools and were further validated by reverse transcription-polymerase chain reaction and receiver operator characteristic (ROC) curve analysis. In the present study, 6 miRNAs (hsa-miR-125b-5p, hsa-miR-150-3p, hsa-miR-199a-3p, hsa-miR-584-5p hsa-miR-199a-5p, and hsa-miR-342-5p) were selected for further validation. hsa-miR-584-5p, hsa-miR-342-5p, and hsa-miR-150-5p with area under curve (AUC) values of 0.846, 0.835, and 0.826, respectively, were identified as promising biomarkers of epilepsy. A logistic model combining three miRNAs (hsa-miR-584-5p, hsa-miR-342-5p, and hsa-miR-199a-3p) could achieve an AUC of 0.883 and a six miRNAs model (hsa-miR-342-5p, hsa-miR-584-5p, hsa-miR-150-5p, hsa-miR-125b-5p, hsa-miR-199a-3p, and hsa-miR-199a-5p) could attain an AUC of 0.888. The predicted probability of multiple miRNA panels was evaluated for differentiating between drug-resistant children and drug-responsive children. The AUC of a six-miRNA panel (hsa-miR-342-5p, hsa-miR-584-5p, hsa-miR-150-5p, hsa-miR-125b-5p, hsa-miR-199a-3p, and hsa-miR-199a-5p) reached 0.823. We identified and confirmed plasma sEVs derived miRNA biomarkers that could be considered as potential therapeutic targets for pediatric epilepsy and drug-resistant epilepsy.

## Introduction

Epilepsy is a serious neurological disease affecting the development of children and their quality of life and having an incidence rate of 33/100,000–82/100,000 cases per year (Plevin and Smith, 2019). Based on the new International League against Epilepsy (ILAE) classification of epilepsy, epilepsy is classified into focal epilepsy, generalized epilepsy, combined generalized, and unknown. Techniques, including neuroimaging and electroencephalography (EEG), contribute to optimizing the classification (Scheffer et al., 2017). Multiple factors such as environmental factors and gene mutations are considered to contribute to epilepsy; thus, the traditional classification of epilepsy based on clinical and neurological history, EEG, and neuroimaging is not sufficient to elucidate its pathogenesis and epileptogenesis (Manford, 2017). Therefore, biomarkers of epileptogenesis need to be identified.

In recent years, more than 20 antiepileptic drugs (AEDs) have been used for epilepsy treatment; however, 20–30% of patients are resistant to AEDs (Chakraborty et al., 2020). Although several new antiepileptic drugs have been approved for children with epilepsy, the incidence rate of drug-resistant epilepsy (DRE) has not been decreased (Li et al., 2013). The risk of injuries, mood and cognitive impairment, and death has increased in children with DRE (Kwan et al., 2011). In addition, no study or clinical trial has been reported on AEDs that can regulate the pathophysiology of seizure suppression, and no biomarkers are available for predicting the response to specific antiepileptic drugs (Barker-Haliski et al., 2015; Ma, 2018). Minimally invasive biomarkers for DRE diagnosis can effectively facilitate disease-related treatment and improve the quality of life.

MicroRNAs (miRNAs) belong to a large family of small (21 nucleotides), non-coding, and single-stranded RNAs functioning as post-transcriptional regulators of gene expression (Bartel, 2009; Chekulaeva and Filipowicz, 2009). The regulatory role of miRNAs is attributed to the base pairing in the 3′-untranslated region (3′-UTR) of target mRNAs, and protein synthesis is inhibited or mRNA deadenylation and decay is promoted (Krol et al., 2010). Depending on the conserved base pairing between mRNAs and miRNAs, 60% of human proteins are estimated to be directly controlled by miRNAs (Ebert and Sharp, 2012). Functional studies have indicated that miRNAs play a role in a variety of human diseases, including cancer, autoimmune diseases, and neurodegenerative disorders (Catanesi et al., 2020; Hou et al., 2021; Komoll et al., 2021). Critical effects of miRNAs on the central nervous system (CNS) have been identified, and numerous studies have reported that miRNAs play a key role in CNS functions and disorders by mediating neurogenesis, neural differentiation, and synaptic plasticity (Krol et al., 2010; Jin et al., 2013; Cukovic et al., 2021). Various miRNAs have been detected in human body fluids, including plasma or plasma, urine, and saliva (Weber et al., 2010; François et al., 2019; Putkonen et al., 2020). Circulating miRNAs detected in various disorders, especially in tumors and cardiovascular diseases, are now considered attractive candidate biomarkers (Ding et al., 2019; Fehlmann et al., 2020; Park et al., 2020). Recently, circulating miRNAs have been investigated in various CNS disorders, including Alzheimer’s disease (Hajjri et al., 2020), Parkinson’s disease (Sancandi et al., 2020), amyotrophic lateral sclerosis (Saucier et al., 2019), major depression disorder (van den Berg et al., 2020), and epilepsy (Chen et al., 2020; Leontariti et al., 2020) for clinical diagnosis and therapeutic assessments. Based on the accessibility and increased stability of circulating miRNAs, we hypothesized that circulating miRNAs could be considered ideal biomarker candidates for predicting the prognosis of epilepsy and drug-resistant epilepsy.

Small extracellular vesicles (sEVs) form a subset of extracellular membrane lipid vesicles released from cells during multivesicular body-plasma membrane fusion with a size ranging from 50 to 150 nm (Tran et al., 2020). sEVs are the carriers of various molecules containing functional proteins, mRNAs, miRNAs, and lipids, which can directly target cells and establish cell-to-cell communication. sEVs as vehicles carrying miRNAs can provide a stable environment for protecting the miRNAs from RNase-dependent degradation compared with freely circulating miRNAs (Gurunathan et al., 2019). sEVs are widely distributed in body fluids, including blood, cerebrospinal fluid, and urine (Pieragostino et al., 2018; Karlsen et al., 2019; Vitorino et al., 2021; Wu et al., 2021). Several studies have reported that sEVs carrying miRNAs in the CNS can be detected in the cerebrospinal fluid that can traverse the blood–brain barrier and come into peripheral circulation; hence, miRNAs in circulating sEVs have potential applications as non-invasive diagnostic and prognosis biomarkers for CNS disorders (Liu et al., 2019; Xia et al., 2019). Therefore, we considered that circulating miRNAs in plasma sEVs can be used as a set of potential biomarkers for epilepsy and pediatric DRE for early diagnosis and treatment optimization.

In this research, we aimed to characterize the expression characteristics of miRNAs extracted from the plasma sEVs of epileptic children (including drug-responsive and drug-resistant) and healthy children using bioinformatics tools. The plasma sEVs derived miRNA biomarkers could be considered as potential therapeutic targets for pediatric epilepsy and drug-resistant epilepsy.

## Materials and Methods

### Study Outline and Patient Characteristics

The study was performed in Zhejiang University School of Medicine, and ethics approval for this experiment was required by the Ethics Committee of the Children’s Hospital of Zhejiang University School of Medicine (2021-IRB-129). Written informed consent was gained from the parents or guardians prior to acquirement of any data.

A total of 90 children clinically diagnosed with epilepsy (46 with drug-responsive epilepsy and 44 with drug-resistant epilepsy) and 37 HCs matched for age and gender were enrolled in this research. Expression of circulating miRNAs from plasma sEVs between epileptic children and healthy children was assessed in this multiphase case-control study. In a discovery phase of the study, plasma samples of 25 epileptic children (12 drug-responsive epileptic and 13 drug-resistant epileptic children) and 10 HCs were pooled using Illumina NovaSeq 6000 technology to evaluate miRNA expression between epileptic children and controls. Subsequently, the levels of differentially expressed plasma sEVs derived circulating miRNAs from additional 65 epileptic children (34 drug-responsive epileptic and 31 drug-resistant epileptic children) and 27 HCs were verified by real-time reverse transcription-polymerase chain reaction (qRT-PCR).

All epileptic children were diagnosed in the Department of Neurology, Children’s Hospital of Zhejiang University School of Medicine. Case inclusion criteria were as follows: (a) Drug-responsive epilepsy was defined as a seizure-free period of a minimum of three times the longest interictal period or 12 months, whereas DRE was characterized by the failure of adequate administration of two AEDs at optimal doses to obtain sustained seizure freedom in the last 12 months by experienced clinicians according to new ILAE classification of epilepsy in 2017 (Kwan et al., 2010; Scheffer et al., 2017); (b) patients with no systemic infection or CNS infection 1 month before blood collection; (c) patients with no febrile convulsions, severe organ diseases, progressive nervous diseases, autoimmune diseases, mental disorders, diabetes, or major congenital neurological diseases; (d) patients with normal blood routine examination; (e) patients with no generalized convulsive seizures within 2 days before collection of blood samples (Shen et al., 2019); (f) patients with no epilepsy surgery before sample harvested. The HCs were recruited by the Examination Center of Children’s Hospital, Zhejiang University School of Medicine, and had no neurological illness, metabolic diseases, and infectious diseases. The hemolyzed samples were excluded from the study.

### Blood Collection and Isolation of Small Extracellular Vesicles

Combination size-exclusion chromatography (SEC) with ultrafiltration was referred to as a low recovery and high specificity method for the isolation of sEVs (Jang et al., 2013; Karimi et al., 2018; Théry et al., 2018). In our study, peripheral blood samples from refractory epileptic children, drug-responsive epileptic children, and HCs were collected in EDTA tubes, followed by centrifugation at 3,000 g for 15 min at 4°C and separation of plasma, and the tubes were stored at −80°C for the following experiments. The plasma sEVs were separated by SEC with slight modifications (Boing et al., 2014). Briefly, 1 mL of the 0.8-μm membrane-filtered plasma samples was overlaid on Exosupur^®^ columns (Echobiotech, China). The columns were further eluted using PBS, and a total amount of 2 mL from eluting fractions was collected in consistent with the manufacturer’s instructions. The fractions were gathered by filtering through a 100-kD molecular weight cutoff spin column of Amicon^®^ Ultra spin filters (Merck, Germany) to a final volume of 200 μL.

### Nanoparticle Tracking Analysis

Vesicles in the suspension liquid with concentrations ranging from 1 × 10^7^/mL to 1 × 10^9^/mL were assessed using ZetaView PMX 110 (Particle Metrix, Meerbusch, Germany) armed with a 405-nm laser source to confirm the size and quantity of the separated vesicles. A video of 60 s was recorded in 30 frames per second, and vesicle movements were tracked using an NTA software (ZetaView 8.02.28).

### Transmission Electron Microscopy

A total amount of 10 μL of plasma sEV samples was kept on a copper mesh for incubation at 20 degrees Celsius for 1 min. Then plasma sEV samples were treated using uranyl acetate solution for 1 min and then washed with sterile distilled water. The water was removed from the samples, and the samples were kept under incandescent light for 2 min. The copper mesh was monitored, and images were captured using a transmission electron microscope (H-7650, Hitachi Ltd., Tokyo, Japan).

### Western Blot Assay

5X sodium dodecyl sulfate (SDS) buffer was used to denature the sEV supernatant at 95°C for 5 min and subject to western blot analysis (10% SDS-polyacrylamide gel electrophoresis; 50 μg of protein per lane). The primary antibodies CD63 (sc-5275, Santa Cruz, CA, United States), Alix (sc-53540, Santa Cruz, CA, United States), TSG101 (sc-13611, Santa Cruz, CA, United States), and calnexin (10427–2-AP, Promega, Madison, WI) were used to probe specific proteins. The cell lysates derived from mesenchymal stem cell (MSC) were used as control.

### Library Preparation and Sequencing

Total RNA samples were extracted and purified from sEVs by the miRNeasy^®^ Mini kit (Qiagen, cat. No. 217004) in accordance with the manufacturer’s instructions. The concentration and purity of RNA samples were evaluated by RNA Nano 6000 Assay Kit of Agilent Bioanalyzer 2100 System (Agilent Technologies, CA, United States). For small RNA libraries, a total of 1–500 ng of RNA per sample was prepared as the input material. Sequencing libraries were constructed using QIAseq miRNA Library Kit (Qiagen, Frederick, MD) according to the manufacturer’s instructions. Reverse transcription (RT) primers with unique molecular indices (UMIs) were introduced to analyze the quantification of miRNA expressions during cDNA synthesis and PCR amplification. Agilent Bioanalyzer 2100 and qPCR technique was used to assess the library quality. The clustering analysis of the index-coded products was accomplished using acBot Cluster Generation System and TruSeq PE Cluster Kitv3-cBot-HS (Illumina, San Diego, CA, United States) in consistent with the manufacturer’s protocol. Preparations of the library were analyzed using an Illumina Hiseq system following cluster production, and paired-end reads were produced.

### Bioinformatics Identification of miRNA

The high-quality sequences were mapped with Silva database, GtRNAdb database, Rfam database and Repbase database through Bowtie software. rRNA, tRNA, small nuclear RNA (snRNA), small nucleolar RNA (snoRNA), other non-coding RNAs, and repeat sequences were refined. The excess reads were applied to exam miRNAs predicted in comparison to known miRNAs of miRbase and Human Genome (GRCh38). Expression matrix of quantified UMI counts of miRNAs was normalized to counts per million (CPM) and calculated to relative log expression via the Edge R package.

### Gene Ontology and Kyoto Encyclopedia of Genes and Genomes Pathway Enrichment Analysis

Based on gene sequence of miRNA and mRNA, both of miRanda (v3.3) and RNAhybrid (v2.1.1) were carried out for confirming the rules of interaction between miRNAs and their targets. GO enrichment analysis of the target genes of differentially expressed miRNAs (DEMs) was performed using the top Gene Ontology (GO) R packages. The database resource, Kyoto Encyclopedia of Genes and Genomes (KEGG) (Kanehisa et al., 2004) refers to awareness of advanced functions and utilizations of the biological system including the cell, the organism, and also the ecosystem, from large-scale datasets even at molecular level constructed using high-throughput experimental techniques like genome sequencing^1^ The KOBAS (Mao et al., 2005) software was served to determine differential enrichment with statistically significance of genes related to KEGG pathways.

### qRT-PCR Analysis of miRNA Expression

Total plasma sEV-miRNA samples were isolated and refined by an miRNeasy^®^ Mini kit (Qiagen, cat. 217,004). The total RNA products were then reverse-transcribed into complementary DNA (cDNA) by PrimeScript™ RT reagent Kit (Perfect Real Time) (TAKARA, RR037A). TaqMan^®^ probe detected the expression level of target gene expression depending on qPCR. A total of 2 μL of cDNA was added to each PCR sample as a template. The primers and probes sequences are presented in Supplementary Table 1. The relative quantification was calculated and normalized to U6 snRNA (internal control) and calculated by the −ΔΔCt method.

### Statistical Analysis

Student’s *t*-tests were used to compare the results for clinical data between patients and controls using SPSS 28.0.1 software. The expression levels of plasma sEVs-miRNA were calculated with the -ΔΔCt method, Student’s *t*-test analysis for significance of *p*-value based on Compare Means of ggpubr package. Logistic regression analysis of qRT-PCR analysis results was executed using Generalized Linear Model in R 3.2.3.^2^ Three or six genes were included for constructing Classification Model. pROC package was used to display and analyze receiver operator characteristic (ROC) curve. Then area under curve (AUC) values were calculated to evaluate the performances of our methods. *P* < 0.05 was considered significant.

## Results

### Clinical Features of Participants

**FIGURE 1:**
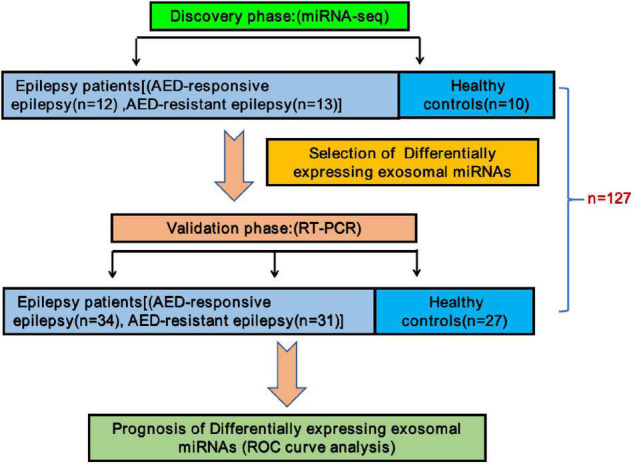
Schematic description of the experimental design. In the discovery phase, a total number of 12 antiepileptic drugs (AED)-responsive children and 13 AED-resistant children were enrolled in this study along with 10 healthy children as controls for miRNA-seq. Additional 92 children (34 AED-responsive children, 31 AED-resistant children, and 27 healthy controls) were enrolled for validation by reverse transcription-polymerase chain reaction. Receiver operating characteristic curves analysis was performed for assessing these sEVs derived miRNAs as potential new diagnostic biomarkers.

A total of 127 participants including 25 epileptic children (12 drug-responsive epileptic and 13 drug-resistant epileptic children) and 10 HCs in the discovery phase and 65 epileptic children (34 drug-responsive epileptic and 31 drug-resistant epileptic children) and 27 HCs in the validation phase of the study were recruited in this study. The study design is presented in Figure 1. The clinical characteristics of individuals are summarized in Table 1. In the validation phase, the mean age at first seizure was 3.34 and 1.73 years, and average of epilepsy duration was 3.09 and 4.86 years in drug-responsive and drug-resistant patients, respectively. Supplementary Table 2 presents that the drug-responsive and drug-resistant children in the validation phase received a median number of 1.32 and 4.19 AED treatment and depakine (77.4%), topiramate (51.6%), oxcarbazepine (51.6%), levetiracetam (48.4%), lacosamide (38.7%), lamotrigine (35.5%), and nitrazepam (35.5%), being the most frequently used in drug-resistant children.

**TABLE 1:** Clinical characteristics features of individuals.

	Discovery set			Large-scale validation set		
	Epilepsy		HCs	Epilepsy		HCs
	AED-r	AED-R		AED-r	AED-R	
NO	12	13	10	34	31	27
Female: Male	7:5	7:6	5:5	18:16	17:14	15:12
Age, Mean ± SD (median)	6.17 ± 3.94 (5.29)	5.49 ± 3.49 (4.75)	6.98 ± 4.39 (6.5)	6.41 ± 3.69 (5.96)	6.63 ± 3.97 (6.41)	6.60 ± 4.05 (6.41)
Epilepsy duration, median (range) (y)	2.52 ± 2.61 (1.52)	2.71 ± 1.81 (2.50)	NA	3.09 ± 2.57 (2.25)	4.86 ± 3.38 (3.83)	NA
Age at first seizure ± SD (median)	3.45 ± 3.32 (2.42)	2.72 ± 3.68 (0.83)	NA	3.34 ± 3.05 (2.37)	1.73 ± 1.60 (1.25)	NA
Number of AEDs used	1.25 ± 0.45 (1)	4.31 ± 0.75 (4)	NA	1.32 ± 0.47 (1)	4.19 ± 0.87 (4)	NA

Age, age at epilepsy onset, duration of epilepsy and number of AEDs used were presented as mean ± standard deviation, rest of data were presented as amount. AED-r, AED-responsive; AED-R, AED-resistant; HCs, Healthy controls; NA, Not Available.

### Characterization of Small Extracellular Vesicles From Human Plasma

**FIGURE 2:**
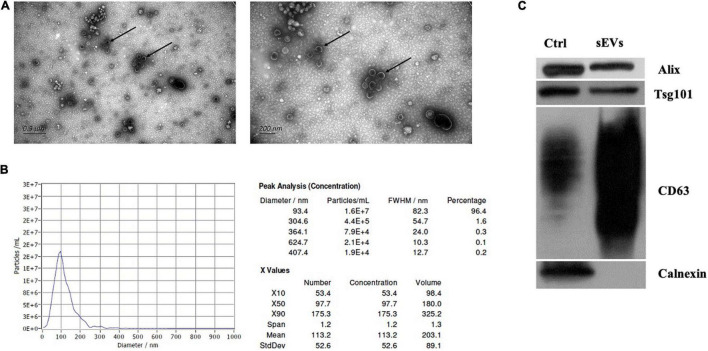
Characterization of sEVs isolated from children’s plasma. **(A)** Morphological analysis of isolated particles by negative stain transmission electron microscopy. Scale bars represent 0.9 μm on the left side and 200 nm on the right side. **(B)** Phenotypic analysis of the samples. The particle size and concentration of isolated particles from the plasma were measured. The curve blue line in the graphs depicts the relationship between particle number (left *Y*-axis) and particle size distribution (nm) of the obtained isolates. The data on the right side represents the particle size and concentration. **(C)** Western blot analysis. The cell lysates of mesenchymal stem cell (MSC) were separated as control. Established marker proteins in lysates such as Alix, Tsg101, and CD63 were detected. Calnexin was also examined as a loading control.

The sEV-enriched fractions were separated from the plasma of all epileptic children and controls by SEC. Transmission electron microscopy (TEM) and nanoparticle tracking analysis (NTA) approaches were used for evaluating the morphology and size distribution of these vesicles. The analysis results indicated that the size of the majority of sEVs ranged from 75 to 200 nm (Figures 2A,B). In sEV-enriched fractions from the plasma, CD63, TSG101, and Alix, which are positive sEV markers, were detected by western blot assay. In contrast, calnexin was undetectable in our sEV-enriched fractions, which served as a negative marker of sEVs (Figure 2C). Our results indicated that the circulating sEVs were successfully isolated from the peripheral blood samples.

### Expressional Changes in Small Extracellular Vesicles Derived miRNAs From Epileptic Children and Healthy Controls

**FIGURE 3:**
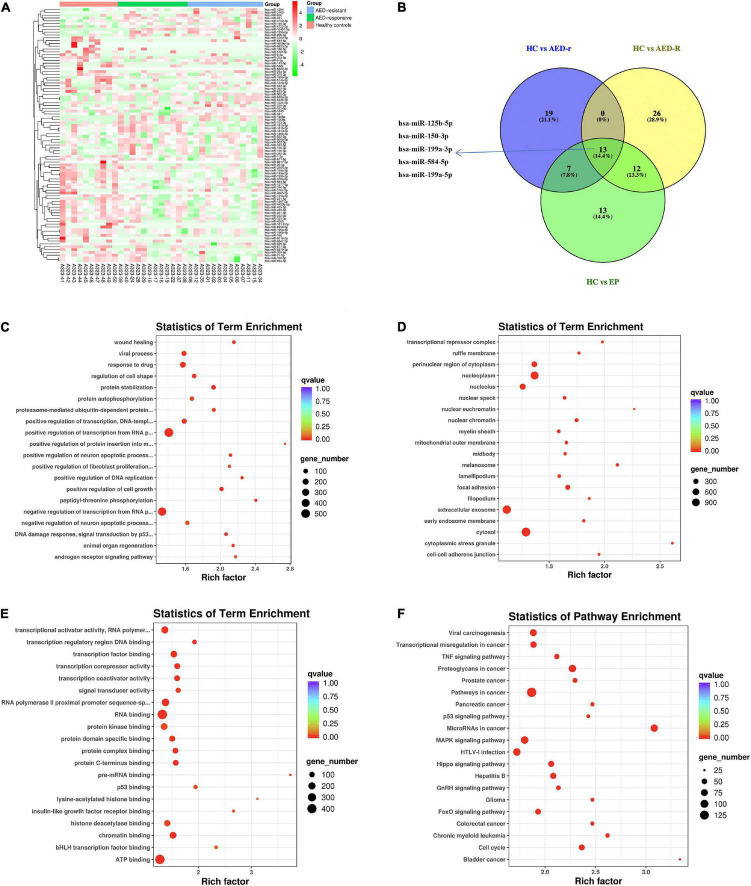
Plasma sEVs derived miRNA profile of epileptic patients. **(A)** Heat map analysis of differentially expressed miRNAs (DEM) levels. The color bar on the right side represents the scale for the Z-score. The red color indicates high expression and the green color indicates low expression. Representative microRNAs are shown on the right side of the heat map. **(B)** The Venn-diagram for the three comparisons [healthy controls (HCs) vs. epilepsy children (EP), HCs vs. antiepileptic drugs (AED)-responsive epilepsy patients (AED-r), and HCs vs. AED-resistant epilepsy patients (AED-R)]. The coincident part indicates DEMs shared between the three comparisons. **(C–E)** The bubble plot shows Gene Ontology terms (Biological Process, Cellular Component, and Molecular Function) of mRNAs targeted by all 90 DEMs in these three groups, *Q*-value is depicted as color code. Bubble size indicates the number of DEVs associated with each term. **(F)** The bubble plot of KEGG pathways enriched in all 90 DEMs. The *Q*-value is depicted as a color code. Bubble size indicates the number of genes associated with each pathway.

**FIGURE 4:**
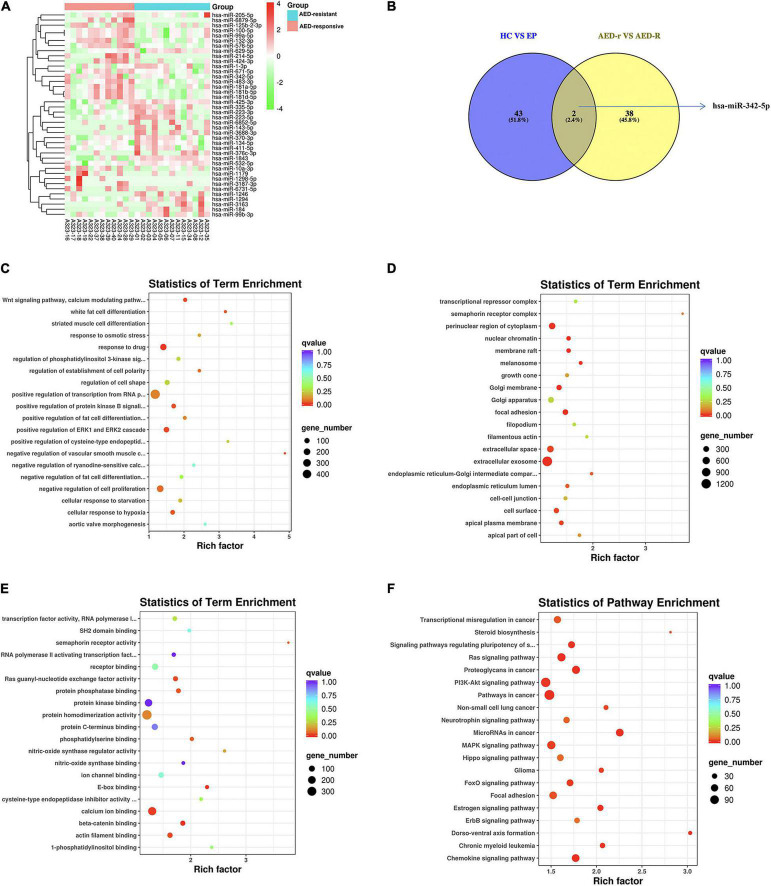
Identification of differentially expressed miRNAs (DEMs) between antiepileptic drugs (AED)-responsive children and AED-resistant children. **(A)** Heat map analysis of the DEMs signature between AED-responsive and AED-resistant epileptic children. The color bar on the right side represents the scale for the Z-score. The red color indicates high expression, and the green color indicates low expression. 40 known DEMs between AED-resistant epilepsy and AED-responsive epilepsy are shown on the right side of the heat map. **(B)** The Venn-diagram for the two comparisons (healthy controls (HCs) vs. epilepsy children and AED-responsive epilepsy patients vs. AED-resistant epilepsy patients). The coincident part indicates DEMs shared between the two comparisons. **(C)** The bubble plot of Gene Ontology (GO) terms (Biological Process) of mRNAs targeted by the DEMs. **(D)** The bubble plot of GO terms (Cellular Component) of mRNAs targeted by the DEMs. **(E)** The bubble plot of GO terms (molecular function) of mRNAs targeted by the DEMs. Bubble size indicates the number of genes associated with each term. **(F)** Bubble plot of KEGG pathway enrichment analyses. The *q*-value is depicted as color code. Bubble size indicates the number of DEMs associated with each pathway.

To investigate the potentially remarkable changes in the sEVs derived miRNAs from the plasma of the epileptic children and controls, the plasma samples of 25 epileptic children (12 drug-responsive epileptic and 13 drug-resistant epileptic children) and 10 HCs were first sequenced by miRNA-sequencing using an Illumina Hiseq platform and approximately 21.61 M raw reads were produced for each sample. A total of 1,318 known miRNAs were detected by the sequencing. To avoid bias resulted from relatively low expression of miRNAs, miRNAs with a median TPM of less than 10 were removed from the subsequent analysis. Screening conditions for DEM analysis were (| log_2_(FC)| > 0.584 and p-adjust < 0.05). We found that the levels of 14 miRNAs were increased, whereas that of 46 miRNAs were decreased in epileptic children in comparison to the healthy group; the levels of 19 miRNAs were increased, and that of 46 miRNAs were decreased in the drug-resistant epileptic children in comparison to the healthy group; the levels of 19 miRNAs were increased, and that of 28 miRNAs were decreased in the drug-responsive epileptic children in comparison to the healthy group. Thus, 90 DEMs were acquired from the union of sEVs derived miRNAs with differential expression in HC vs. drug-responsive epilepsy, HC vs. drug-resistant epilepsy and HC vs. epilepsy (Figure 3A). We performed GO term and KEGG analysis for determining the potential functions of 90 DEMs. GO term analysis and KEGG analysis showed that 5398 mRNAs targeted by 90 DEMs were enriched in the cytosol, nucleoplasm, negative regulation of RNA, RNA binding, ATP binding, MAPK signaling pathway, and Hippo signaling pathway (Figures 3C–F). A total of 13 known DEMs were detected from the intersection of these three groups (Figure 3B) and selected for further validation. The sequences of these 13 microRNAs are presented in Supplementary Table 3. The expression of 22 miRNAs increased and the expression of another 25 miRNAs decreased in the drug-resistant epileptic children compared with drug-responsive epileptic children (Figure 4A), two known DEMs (hsa-miR-342-5p and hsa-miR-1294) were detected from the intersection of epileptic children vs. HCs and drug-resistant epileptic children vs. drug-responsive epileptic children at the same time (Figure 4B). GO term analysis and KEGG analysis found that mRNAs targeted by 47 DEMs significantly enriched in sEVs, perinuclear region of cytoplasm, calcium ion binding, Ras signaling pathway, PI3K-Akt-signaling pathway, MAPK signaling pathway, and chemokine signaling pathway (Figures 4C–F). Hsa-miR-342-5p and hsa-miR-1294 were also elected for further verification.

### Small Extracellular Vesicles Derived miRNAs as Diagnostic Biomarkers for Epilepsy in the Validation Phase

**FIGURE 5:**
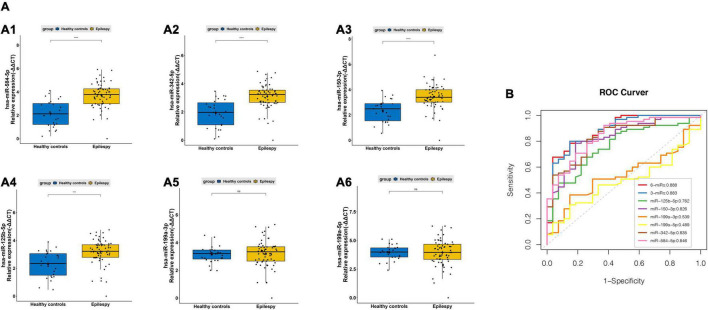
PCR validation of sEVs derived miRNAs between the pediatric epilepsy and controls. **(A)** The relative expression level of sEVs derived hsa-miR-584-5p **(A1)**, hsa-miR-342-5p **(A2)**, miR-150-3p **(A3)**, hsa-miR-125b-5p **(A4)**, hsa-miR-199a-3p **(A5)** and hsa-miR-199a-5p **(A6)** measured by PCR in 92 independent validation samples (epilepsy children vs. HCs) (average of expression ± SD; *t*-test, ^***^p < 0.001, ^*⁣*⁣**^p < 0.0001). **(B)** ROC curves to validate the diagnosis efficiency (epilepsy children vs. HCs) of sEVs derived hsa-miR-584-5p, hsa-miR-342-5p, miR-150-3p, hsa-miR-125b-5p, hsa-miR-199a-3p and hsa-miR-199a-5p, three miRNAs (hsa-miR-584-5p, hsa-miR-342-5p, and hsa-miR-199a-3p) combination (AUC = 0.883), and six miRNAs combination (AUC = 0.888).

In the screening phase, we found five top miRNAs (hsa-miR-199a-3p, hsa-miR-125b-5p, hsa-miR-150-3p, hsa-miR-584-5p, and hsa-miR-199a-5p) of 13 DEMs exhibited most significant difference with a median TPM of more than fifty between the epileptic children and controls (Figure 3B), while hsa-miR-342-5p demonstrated higher median TPM compared to hsa-miR-1294 in discriminating epileptic children from HCs, and drug-resistant epileptic children from drug-responsive epileptic children at the same time. To identify potential biomarkers for pediatric epilepsy, five miRNAs (hsa-miR-199a-3p, hsa-miR-125b-5p, hsa-miR-150-3p, hsa- hsa-miR-584-5p, and hsa-miR-199a-5p) and hsa-miR-342-5p were selected from 13 DEMs for further validation (Figures 3B, 4B). The levels of miRNAs were determined in a band of 65 epileptic patients (34 drug-responsive epileptic and 31 drug-resistant epileptic patients) and 27 HCs in the validation phase. miRNA levels were normalized to U6 snRNA (internal control) and calculated by the -ΔΔCt method (Figure 5A). All experiments were performed in triplicates and their mean value was calculated. We found hsa-miR-584a-5p, hsa-miR-342a-5p, hsa-miR-150-3p, and hsa-miR-125b-5p had significantly different levels (*P* < 0.0001) in epileptic patients than in the health controls. Among these miRNAs, hsa-miR-584a-5p had the highest diagnostic accuracy with an area under the ROC curve of 0.846 (95% CI: 0.761–0.931) with a sensitivity of 92.3% and a specificity of 63%; hsa-miR-342a-5p showed the diagnostic accuracy with an AUC of 0.835 (95% CI: 0.747–0.923) with a sensitivity of 90.8% and a specificity of 63.0%; Subsequently, multiple miRNA panels were evaluated for differentiating epileptic patients from health children. A logistic model combining three miRNAs (hsa-miR-584-5p, hsa-miR-342-5p, and hsa-miR-199a-3p) attained an AUC of 0.883 with specificity 0.969 and sensitivity 0.556, and a six miRNAs model (hsa-miR-342-5p, hsa-miR-584-5p, hsa-miR-150-5p, hsa-miR-125b-5p, hsa-miR-199a-3p, and hsa-miR-199a-5p) achieved an AUC of 0.888 (Figure 5B).

### Diagnostic Efficacy of Small Extracellular Vesicles Derived miRNAs Between Drug-Responsive Epilepsy and Drug-Resistant Epilepsy in the Validation Phase

**FIGURE 6:**
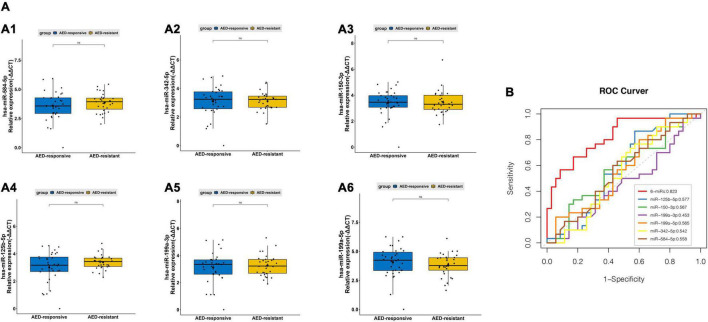
PCR validation of sEVs derived miRNAsbetween the pediatric drug-responsive epilepsy and drug-resistant epilepsy. **(A)** The relative expression level of sEVs derived hsa-miR-342-5p **(A1)**, has-miR-584-5p **(A2)**, miR-150-3p **(A3)**, hsa-miR-125b-5p **(A4)**, hsa-miR-199a-3p **(A5)** and has-miR-199a-5p **(A6)** measured by PCR in 65 independent validation samples (average of expression ± standard deviation (SD); *t*-test, ns *p* > 0.05). **(B)** ROC curves to validate the diagnosis efficiency (AED-responsive epilepsy patients vs. AED-resistant epilepsy patients) of sEVs derived hsa-miR-342-5p, hsa-miR-584-5p, miR-150-3p, hsa-miR-125b-5p, hsa-miR-199a-3p and hsa-miR-199a-5p and their combinations (AUC = 0.823).

Delayed and inaccurate diagnosis of pediatric DRE cause great difficulties in treatment and prognosis. Here, for seeking diagnosis markers in separating pediatric epilepsy from pediatric drug-resistant epilepsy, six miRNAs (hsa-miR-342-5p, hsa-miR-584-5p, hsa-miR-150-3p, hsa-miR-125b-5p, hsa-miR-199a-3p, and hsa-miR-199a-5p) (Figures 3B, 4B) were picked for further examination. In our study, these miRNAs have slightly differential expression level between drug-responsive epilepsy and drug-resistant epilepsy in the validation phase (Figure 6A). Hence, for improving diagnostic efficacy of these miRNAs, the predicted probability of multiple miRNA panels was evaluated. The results indicated a combination of sEVs isolated miRNAs showed significantly higher accuracy in differentiating between drug-resistant children and drug-responsive children. The AUC of a six-miRNA panel (hsa-miR-342-5p, hsa-miR-584-5p, hsa-miR-150-3p, hsa-miR-125b-5p, hsa-miR-199a-3p, and hsa-miR-199a-5p) reached 0.823 with a sensitivity of 96.7% and a specificity of 54.3% (Figure 6B).

## Discussion

Accurate and non-invasive diagnosis is of great importance for proposing efficient therapeutic strategies. Novel biomarkers of epileptogenesis need to be identified for predicting the prognosis of pediatric DRE that can effectively promote disease-related treatment. A set of circulating miRNAs such as miR-146a, miR-134, hsa-miR-199a-3p, hsa-miR-335-5p, hsa-miR-126-5p, hsa-miR-27a-3p, miR-142, miR-3613-5p, hsa-miR-328-3p, and miR-223 that represents potential molecular biomarkers of epilepsy has already been widely studied and deeply explored in adult epileptic patients (Yan et al., 2017; Ma, 2018; Raoof et al., 2018; Huang et al., 2020; Leontariti et al., 2020; De Benedittis et al., 2021). In recent years, the role of circulating sEVs derived miRNAs with a stable environment for protecting miRNAs from RNase-dependent degradation as more suitable biomarkers is evident for the CNS. A recent study has reported that the circulating miRNAs from sEVs have implications as diagnostic and prognostic biomarkers for structural epilepsies (Chen et al., 2020).

In the present study, RNA-seq analysis was performed to identify sEVs derived miRNAs from the plasma samples of pediatric epileptic patients. To the best of our knowledge, this study is the first to determine the expression profile of plasma sEVs derived miRNAs from pediatric epileptic patients using next-generation sequencing. In the discovery phase, DEMs were analyzed in epileptic children compared with controls, in drug-resistant epileptic children compared with controls, and in drug-responsive epileptic children compared with controls. On the basis of bioinformatics analysis, 6 DEMs (hsa-miR-199a-3p, hsa-miR-125b-5p, hsa-miR-150-3p, hsa-miR-584-5p, hsa-miR-199a-5p, and hsa-miR-342a-5p) were finally selected for validation by RT-qPCR. Finally, sEVs derived hsa-miR-584a-5p, hsa-miR-342a-5p, hsa-miR-150-3p, and hsa-miR-125b-5p were identified as potential biomarkers that can distinguish between patients with epilepsy and HCs with an AUC of 0.846, 0.835, 0.826, and 0.762, respectively.

Drug resistance is a key challenge in all epilepsy types. Nevertheless, the variations and seizures involved in epilepsy complicate the diagnosis of drug-resistant epilepsy. Delayed and inaccurate diagnosis prevents timely and appropriate treatment, thus greatly afflicting patients with adverse outcomes and economic burden. Unfortunately, our study failed to identify a suitable biomarker alone used for predicting both pediatric epilepsy and drug-resistant epilepsy. Strikingly, multiple plasma sEVs derived miRNAs showed good performance in identifying drug-resistant pediatric patients (Figure 6B). Furthermore, 47 DEMs have been identified in patients with drug-resistant epilepsy rather than patients with drug-responsive epilepsy, and this provides a good direction for our follow-up study on drug-resistant epilepsy (data not shown).

Decreased levels of Hsa-miR-125b-5p and hsa-miR-150 have been reported in rat models at different time points after status epileptics (Risbud and Porter, 2013). Hsa-miR-342-5p level was significantly decreased in plasma samples from drug-resistant patients compared with the healthy group, but pediatric patients were not separated for further study (Wang et al., 2015). The variable levels of hsa-miR-199a between healthy and epileptic groups showed the potential of hsa-miR-199a as a diagnostic marker (Ma, 2018). The role of Hsa-miR-584-5p in medulloblastoma progression and chemotherapy is evident (Abdelfattah et al., 2018). This is the first study to report the excellent behavior of hsa-miR-584-5p in epilepsy diagnosis.

We also analyzed the latent function of plasma sEVs derived miRNAs that were upregulated/downregulated in epilepsy, including drug-responsive epilepsy and drug-resistant epilepsy. GO analysis and KEGG pathway enrichment analysis indicated that mRNAs targeted by DEMs were enriched in the cytosol, and negative regulation of RNA, protein homodimerization activity, and Ras signaling pathways might be considered potential treatment targets for epilepsy.

## Conclusion

We measured the expression profiles of the miRNAs derived from plasma sEVs in epileptic children, including drug-responsive epileptic children and drug-resistant epileptic children. Our findings suggested that plasma sEVs derived miRNAs might be considered predictive targets as a minimally invasive method for pediatric epilepsy prognosis, and multiple plasma sEVs derived miRNA panels can be used for distinguishing between drug-resistant children and drug-responsive children.

## Data Availability Statement

The data presented in the study are deposited in the GEO repository, accession number GSE193842.

## Ethics Statement

The studies involving human participants were reviewed and approved by the Ethics Committee of the Children’s Hospital of Zhejiang University School of Medicine (2021-IRB-129). Written informed consent to participate in this study was provided by the participants’ legal guardian/next of kin.

## Author Contributions

FG and YLW designed the data collection instruments, collected the data, performed the initial analyses, reviewed and revised the manuscript, and conceptualized and designed the study. YPW, YC, LX, MYZ, CYZ, WRZ, GXS, LL, PFJ, ZFY, and ZYZ coordinated and supervised data collection, and critically reviewed the manuscript for important intellectual content. All authors contributed to the article and approved the submitted version.

## Conflict of Interest

The authors declare that the research was conducted in the absence of any commercial or financial relationships that could be construed as a potential conflict of interest.

## Publisher’s Note

All claims expressed in this article are solely those of the authors and do not necessarily represent those of their affiliated organizations, or those of the publisher, the editors and the reviewers. Any product that may be evaluated in this article, or claim that may be made by its manufacturer, is not guaranteed or endorsed by the publisher.

## Footnotes

1
http://www.genome.jp/kegg/

2
www.r-project.org
