# Supplementary material for: Circulating MicroRNAs From Plasma Small Extracellular Vesicles as Potential Diagnostic Biomarkers in Pediatric Epilepsy and Drug-Resistant Epilepsy
Source: Front Mol Neurosci. 2022 Feb 10;15:823802. doi: 10.3389/fnmol.2022.823802 (PMC8866954; doi:10.3389/fnmol.2022.823802)
Supplement: Supplementary file 1 [file Table_1.DOCX]

**Supplementary Table 1. Sequences of the oligonucleotides used for PCR**

| Primer/probe | Sequence (5ʹ-3ʹ) |
| --- | --- |
| U6-RT | AACGCTTCACGAATTTGCGT |
| U6-S | CTCGCTTCGGCAGCACA |
| U6-A | AACGCTTCACGAATTTGCGT |
| U6 probe | FAM-AGAAGATTAGCATGGCCCCTGCGCA-BHQ1 |
| hsa-miR-199a-3p-RT | GTCGTATCCAGTGCAGGGTCCGAGGTATTCGCACTGGATACGACTAACCAA |
| hsa-miR-199a-3p-F | ACCGACAGTAGTCTGCACA |
| hsa-miR-199a-3p-P | FAM-TTCGCACTGGATACGACTAACCAA-BHQ1 |
| hsa-miR-125b-5p-RT | GTCGTATCCAGTGCAGGGTCCGAGGTATTCGCACTGGATACGACTCACAAG |
| hsa-miR-125b-5p-F | AGCCTCCCTGAGACCCTAA |
| hsa-miR-125b-5p-P | FAM-TTCGCACTGGATACGACTCACAAG-BHQ1 |
| hsa-miR-150-3p-RT | GTCGTATCCAGTGCAGGGTCCGAGGTATTCGCACTGGATACGACCTGTCC |
| hsa-miR-150-3p-F | CGGCTGGTACAGGCCT |
| hsa-miR-150-3p-P | FAM-TTCGCACTGGATACGACCTGTCC-BHQ1 |
| hsa-miR-584-5p-RT | GTCGTATCCAGTGCAGGGTCCGAGGTATTCGCACTGGATACGACCTCAGT |
| hsa-miR-584-5p-F | ACCTTATGGTTTGCCTGGG |
| hsa-miR-584-5p-P | FAM-TCGCACTGGATACGACCTCAGT-BHQ1 |
| hsa-miR-199a-5p-RT | GTCGTATCCAGTGCAGGGTCCGAGGTATTCGCACTGGATACGACGAACAG |
| hsa-miR-199a-5p-F | CGCCCAGTGTTCAGACTAC |
| hsa-miR-199a-5p-P | FAM-TTCGCACTGGATACGACGAACAG-BHQ1 |
| hsa-miR-342-5p-RT | GTCGTATCCAGTGCAGGGTCCGAGGTATTCGCACTGGATACGACTCAATC |
| hsa-miR-342-5p-F | GGCGAGGGGTGCTATCT |
| hsa-miR-342-5p-P | FAM-TTCGCACTGGATACGACTCAATCAC-BHQ1 |
