# Supplementary material for: Circulating MicroRNAs From Plasma Small Extracellular Vesicles as Potential Diagnostic Biomarkers in Pediatric Epilepsy and Drug-Resistant Epilepsy
Source: Front Mol Neurosci. 2022 Feb 10;15:823802. doi: 10.3389/fnmol.2022.823802 (PMC8866954; doi:10.3389/fnmol.2022.823802)
Supplement: Supplementary file 2 [file Table_2.DOCX]

**Supplementary Table 2. The frequency of administration of each AED**

|  | **Discovery set** | | **Large-scale validation set** | | **Discovery and validation** | |
| --- | --- | --- | --- | --- | --- | --- |
|  | **Epilepsy** | | **Epilepsy** | | **Epilepsy** | |
|  | **AED-responsive** | **AED-resistant** | **AED-responsive** | **AED-resistant** | **AED-responsive** | **AED-resistant** |
| **NO** | **12** | **13** | **34** | **31** | **46** | **44** |
| **Number of AEDs used** | **1.25 ±0.45 (1)** | **4.31±0.75 (4)** | **1.32±0.47 (1)** | **4.19±0.87 (4)** | **1.30 ±0.47 (1)** | **4.23±0.83 (4)** |
| **Depakine** | **5(41.6%)** | **12(92.3%）** | **8(23.5%)** | **24(77.4%)** | **13(28.2%)** | **36(81.2%)** |
| **topiramate** | **2(16.7%)** | **7（53.8%）** | **7(20.6%)** | **16(51.6%)** | **9(19.6%)** | **23(52.3%)** |
| **levetiracetam** | **5(50%)** | **6（46.1%）** | **19(55.9%)** | **15(48.4%)** | **24(52.1%)** | **21(47.7%)** |
| **oxcarbazepine** | **0** | **5（38.4%）** | **8(23.5%)** | **16(51.6%)** | **8(17.4%)** | **21(47.7%)** |
| **Nitrazepam** | **0** | **6（46.1%）** | **0** | **11(35.5%)** | **0** | **17(38.6%)** |
| **perampanel** | **0** | **4（30.7%）** | **0** | **7(22.6%)** | **0** | **11(25.0%)** |
| **clonazepam** | **0** | **3（23.1%）** | **0** | **4(12.9%)** | **0** | **7(15.9%)** |
| **lamotrigine** | **1(8.33%)** | **4（30.7%）** | **0** | **11(35.5%)** | **1(2.2%)** | **15(34.1%)** |
| **Zonisamide** | **0** | **2（15.4%）** | **0** | **1(3.2%)** | **0** | **3(6.8%)** |
| **Lacosamide** | **0** | **3（23.1%）** | **1(2.9%)** | **12(38.7%)** | **1(2.2%)** | **15(34.1%)** |
| **clobazam** | **0** | **1（7.7%）** | **0** | **0** | **0** | **1(2.3%)** |
| **prednisone** | **1(8.33%)** | **1（7.7%）** | **1(2.9%)** | **3(9.7)** | **2(4.4%)** | **4(9.1%)** |
