# Supplementary material for: Circulating MicroRNAs From Plasma Small Extracellular Vesicles as Potential Diagnostic Biomarkers in Pediatric Epilepsy and Drug-Resistant Epilepsy
Source: Front Mol Neurosci. 2022 Feb 10;15:823802. doi: 10.3389/fnmol.2022.823802 (PMC8866954; doi:10.3389/fnmol.2022.823802)
Supplement: Supplementary file 3 [file Table_3.DOCX]

**Supplementary Table 3. Thirteen known DEMs shared among three analyses in RNA-seq analysis (screening phase) organized by P Value.**

|  | EV-miRNAs | Log2 FC | P Value |
| --- | --- | --- | --- |
| **Heathy control versus Epilepsy patients** | hsa-miR-199b-5p | -2.203 | 3.82E-05 |
|  | hsa-miR-10401-3p | 4.103 | 6.89E-05 |
|  | hsa-miR-199a-3p | -0.635 | 3.25E-04 |
|  | hsa-miR-199b-3p | -0.635 | 3.25E-04 |
|  | hsa-miR-125b-5p | 0.6571 | 5.65E-04 |
|  | hsa-miR-1268a | 3.381 | 1.27E-03 |
|  | hsa-miR-6516-3p | -3.431 | 2.10E-03 |
|  | hsa-miR-150-3p | 1.227 | 2.90E-03 |
|  | hsa-miR-421 | 1.294 | 4.45E-03 |
|  | hsa-miR-584-5p | -0.797 | 5.82E-03 |
|  | hsa-miR-199a-5p | -0.927 | 9.41E-03 |
|  | hsa-miR-4732-3p | 1.019 | 1.03E-02 |
|  | hsa-miR-4433b-5p | -0.912 | 1.97E-02 |
